# A taste of one’s own medicine: *Bacillus velezensis* isolated from adult housefly intestines demonstrates effective fly control

**DOI:** 10.3389/fimmu.2025.1575292

**Published:** 2025-09-16

**Authors:** Ying Li, Shumin Wang, Dawei Yao, Kexin Zhang, Yansong Yin, Xinxin Kong, Jinxiao Li, Lingxia Zeng, Ruiling Zhang, Zhong Zhang

**Affiliations:** ^1^ School of Medicine, Nankai University, Tianjin, China; ^2^ School of Clinical and Basic Medical Science, Shandong First Medical University and Shandong Academy of Medical Sciences, Tai’an, Shandong, China; ^3^ Collaborative Innovation Center for the Origin and Control of Emerging Infectious Diseases, Shandong First Medical University and Shandong Academy of Medical Sciences, Tai’an, China; ^4^ School of Life Science, Shandong First Medical University and Shandong Academy of Medical Sciences, Tai’an, China; ^5^ Health Science Center, Xi’an Jiaotong University, Xi’an, China; ^6^ Hospital for Skin Diseases, Shandong First Medical University, Ji’nan, China; ^7^ Shandong Provincial Institute of Dermatology and Venereology, Shandong Academy of Medical Sciences, Ji’nan, China; ^8^ Medical Science and Technology Innovation Center, The First Affiliated Hospital of Shandong First Medical University, Jinan, Shandong, China; ^9^ School of Life Science, Shandong Second Medical University, Weifang, Shandong, China

**Keywords:** housefly larva, gut microbiota, *Bacillus velezensis*, 16S rRNA gene sequencing, transcriptome, bacteria-host interactions

## Abstract

**Introduction:**

*Bacillus* spp. are widely used as biological agents for managing diseases in crops, livestock, poultry, and aquatic animals. *Bacillus velezensis*, a novel species within the *Bacillus* genus, is extensively used in the biological control of animal and plant diseases. However, the association between *B. velezensis* and insect hosts remains a complex and poorly understood process.

**Methods:**

In this study, we utilized a housefly larvae model to investigate the relationship between *B. velezensis* and houseflies by examining the changes in intestinal microbiota, transcriptomics, and humoral immunity following symbiotic *B. velezensis* treatment.

**Results:**

The results revealed striking dynamic changes in the bacterial community composition of larvae in the treatment group at the genus level. Notably, *Providencia* and *Morganella* content increased, while *Enterobacter* content decreased, leading to inhibited larval growth. Moreover, the bacterial association with the larva significantly impacted the larval transcriptome, modulating the expression of genes involved in various biological pathways, including host growth and development, macronutrient metabolism, and energy production, which are essential for insect development and survival. Oral feeding of *B. velezensis* also caused significant morphological changes in the larval gut, resulting in notable larval mortality, cell degeneration, shrinkage, and the formation of various vacuoles. Additionally, we observed a significant decrease in immune response in housefly larvae, with a reduction in phenoloxidase activity and melanization ability in treated larvae compared to controls.

**Discussion:**

Therefore, *B. velezensis* can damage the vital functions of housefly larvae and may be utilized as a microecological regulator for the green prevention and control of housefly populations.

## 1 Introduction

The housefly (*Musca domestica*) is one of the most widespread health pests in the environment and is the dominant species in many locations (1). As a significant vector closely associated with humans, the housefly not only mechanically transmits numerous diseases, including cholera, dysentery, and typhoid fever, but also has a severe impact on the environment. However, infections by pathogenic microorganism can hinder the growth of housefly larvae and cause high mortality, which is crucial for achieving effective prevention and control of these sanitary pests (2, 3).

Entomopathogenic bacteria (EPBs) are microorganisms that produce pest-specific toxins and play a crucial role in global pest insect control (4–10). For instance, the combinations of *Photorhabdus luminescens* with *Bacillus thuringiensis kurstaki* has been shown to inhibit the growth of the African cotton leafworm, *Spodoptera littoralis* (Lepidoptera: Noctuidae) (11). Additionally, *Serratia entomophila* and *Serratia proteomaculans* can induce amber disease in the larvae of the grass grub, *Costelytra zealandica*, which is marked by rapid cessation of feeding, gut clearance, and a prolonged chronic infection phase leading to the death of the host insect (12, 13). Moreover, *Pseudomonas entomophila* is highly pathogenic for both larvae and adults of *Drosophila melanogaster*, triggering a systemic immune response that causes extensive destruction of gut cells in *D. melanogaster* after ingestion (14, 15).

It is well-established that the insect gut harbors a diverse indigenous microbiota (16), which plays a crucial role in various aspects of insect physiology, including nutritional metabolism, development, morphogenesis, immunity, and behavior (17–19). Certain pathogenic bacteria not only exert their own virulence but also destabilizing the gut community upon invasion, potentially leading to various diseases in insects (20). Previous studies have shown that culturable bacteria in the housefly gut significantly influence the growth, development, humoral immunity, and intestinal microbiota diversity of housefly larvae. For instance, high concentrations of *Pseudomonas aeruginosa* disrupted the gut microbiota composition and suppressed the growth of beneficial bacteria, leading to compromised intestinal barrier and, consequently, inhibited development of housefly larvae (21).

*Bacillus* is currently the most extensively studied pathogen (22–25). It is known for producing spores that are resistant to harsh environmental conditions and can form sporophytic crystals with protein toxins during growth and development, which can be highly virulent to various insects, particularly those in the *Lepidoptera* order (26). Presently, the commonly used bacterial insecticides, both domestically and internationally, include *Bacillus thuringiensis*, *Bacillus popilliae*, and *Bacillus* sp*aericus* (22, 27). However, there has been limited research on the effects of *B. velezensis* on insects, and its potential as a novel insecticidal pathogen remains unclear.

To investigate the fly-killing activity of *B. velezensis*, we used housefly larvae as a model for studying gut-associated bacteria-host interactions. A *B. velezensis* strain was isolated from the gut of adult houseflies and applied to the larvae. By feeding the larvae with *B. velezensis*, we analyzed its negative effects on the larvae and explored the pathogenic mechanism. We then assessed the impact of bacterial infection on the gut microbial community composition using 16S rRNA gene sequencing. Additionally, we fed the larvae diets supplemented with high concentrations of *B. velezensis* and observed the effects on their transcriptomic composition. Furthermore, we investigated changes in the innate immunity of the larvae, demonstrating the negative impact of bacterial invasion by observing intestinal damage. This study provides a theoretical foundation and a new strategy for using *B. velezensis* in the prevention and control of houseflies.

## 2 Methods

### 2.1 Sampling, isolation and screening of bacteria

The houseflies were obtained from a colony that has been reared in the Laboratory of Vector and Vector-borne Diseases at Shandong First Medical University since 2005. *B. velezensis* Bv was isolated from the gut of adult houseflies. For long-term storage, the bacterial cultures were preserved at −80°C in glycerol [1:1 (v: v)].

### 2.2 Whole genome sequencing, assembly, and annotation

A single colony of *B. velezensis* was inoculated in NB medium and cultured overnight in a 37°C incubator. The supernatant was discarded, and the bacterial pellet was collected by centrifugation at 12000 r/min for 20 min at 4°C. The pellet was then washed with 1 × PBS buffer, and this procedure was repeated 2 to 3 times until the supernatant was clear. The resulting bacterial pellet was frozen at −80°C and sent to a company for whole genome sequencing of *B. velezensis*. Nanopore sequencing technology was used to construct the DNA library and perform sequencing. The raw sequencing data contained a certain proportion of low-quality reads. To ensure the accuracy and reliability of the analysis, quality control of the data was performed, followed by correction and optimization using second-generation sequencing data. Coding genes were predicted from the assembled genomes using Prokka (Version: 1.14.6). Functional annotation of the protein sequences was completed by BLASTp comparison with the cluster of orthologous groups (COG) and the Kyoto Encyclopedia of Genes and Genomes (KEGG) database, which provides information on gene products and metabolic pathways. The virulence genes of the test strains were analyzed using a BLASTp search of the virulence factor database (Virulence Factors Database, VFDB).

### 2.3 Larvicidal activity of bacteria

*B. velezensis* was inoculated into freshly prepared LB liquid medium and placed in a constant temperature shaker. After shaking at 110 rpm for 24 h at 37°C, the concentration of *B. velezensis* reached 2 × 10^8^ CFU/mL, which was used as the high concentration of *B. velezensis*. The bacterial stock solution was diluted into six concentrations (2 × 10^2^ – 2 × 10^7^) using sterile water in equal proportions. The seven concentrations of *B. velezensis* culture suspensions were mixed with sterilized wheat bran at a 1:1 ratio as feed for housefly larvae in the experimental group. LB was used for the control groups. To ensure air permeability, a 5 mL centrifuge tube with a small hole at the top was used, and an equal amount of wheat bran was placed in each tube. One-day-old larvae were carefully transferred into the centrifuge tubes of each group using a fine brush and sealed with cotton plugs. The tubes were then placed in an artificial climate incubator. The results were observed and recorded every 24 h, and larvae that did not respond to a light touch with a brush were considered dead. Ten housefly larvae were treated with each concentration, with the experiment repeated 10 times, totaling 700 larvae. The number of deaths was counted, and the LD_50_ was calculated according to the Reed-Muench method (28).

### 2.4 Bacterial infection in a housefly larval model

Using the same experimental method described above, the *B. velezensis* stock solution (Ba, 2 × 10^8^ CFU/mL) and its dilution at 10^2^ (Bb, 2 × 10^6^ CFU/mL), 10^4^ (Bc, 2 × 10^4^ CFU/mL), 10^6^ (Bd, 2 × 10^2^ CFU/mL) fold were used for feeding experiments. The details are as follows: Ba, Bb, Bc, and Bd. *B. velezensis* suspensions served as the experimental groups, while LB (Ct) was used as the control groups. *B. velezensis* culture suspensions were mixed with sterilized wheat bran at a 1:1 ratio as feed for housefly larvae in the experimental group. Eight 1^st^ housefly larvae were placed in centrifuge tubes containing equal amounts of wheat bran and were fed for a total of 3 days. Three perforated test tubes were used per group. Every day, five larvae samples were taken from each group of test tubes to measure their weight and length until the third day. At the end of the experiment, the pupal weight, pupation rate, and emergence rate were recorded. Larvae samples were collected from different test tubes daily for 16S rRNA high-throughput sequencing of intestinal flora (29, 30).

### 2.5 Transcriptome assembly and identification of differentially expressed genes

Intestines from housefly larvae in both the treatment (Ba) and control groups (Ct) were randomly selected for transcriptomic analysis. Total RNA from the housefly samples was isolated using TRIzol Reagent (Invitrogen, United States), following the manufacturer’s instructions. The quality and quantity of the RNA were assessed using a NanoPhotometer^®^ spectrophotometer, and RNA integrity was evaluated using an Agilent 4200 TapeStation System. Libraries were prepared using the TruSeq Stranded mRNAseq Sample Prep Kit (Illumina, San Diego, CA), according to the manufacturer’s instructions, and sequenced on an Illumina sequencing platform (Illumina NoveSeq 6000) to generate 150 bp paired end reads. Clean data were obtained by removing sequencing adaptors and poor-quality reads from raw data before transcript assembly. The clean data were then mapped to the *M. domestica* (Diptera: Muscidae) genome (*Musca _domestica* 2.0.2, NCBI, GCF_000371365.1) using STAR (version 2.7.6a) (31) and assembled with StringTie (version 1.3.3). All transcripts were annotated, and their expression levels were quantified using FPKMs. Transcripts with a p-value of < 0.05 and at least a twofold log2(fold change) > 1 or < −1 were considered DEGs and identified using the edgeR (R-3.2.4) package in R. Gene ontology (GO) enrichment analyses of the DEGs were performed using topGO (v2.42.0). KEGG enrichment analyses was conducted with KOBAS 3.0 (32). GO and KEGG pathways with a p-value < 0.05 were considered significantly enriched.

### 2.6 Plate confrontation assay

The specific methods used for this assay are based on previous studies conducted in this laboratory.

In order to elucidate the interactions between *B. velezensis* and the remaining gut bacteria of the housefly (i.e., *Serratia marcescens*, *Proteus stuartii*, *Pseudomonas vermicola*, *Klebsiella pneumoniae*, *Enterobacter hormaechei*, *Enterobacter cloacae*, *Asaia bereziniae*, *Lactobacillus fusiformis*, *Pseudomonas aeruginosa*, *Lactococcus lactis*, and *Bacillus safensis*), plate confrontation assays were performed on nutrient agar (NA) medium plates. Each plate was partitioned into two equal sections; one half was inoculated with *B. velezensis*, while the other half served as the negative control. Sterile filter papers with a diameter of 6 millimeters were positioned on both sides of the agar plates, and 10 μl of *Serratia marcescens* bacterial suspension was dispensed onto the filter paper. For the assessment of other bacterial strains, an identical experimental procedure was employed. All plates were then incubated at 37°C, and the growth diameters of each bacterium were measured after a 24 - hour incubation period. The entire experiment was replicated six times to ensure biological independence and statistical reliability.

### 2.7 Effects of feeding isolated *B. velezensis* on phenoloxidase activity in housefly larvae

The housefly larval feeding experiment spanned three consecutive days, with larval samples collected daily from each experimental group. Each larval sample was transferred into a centrifuge tube filled with phosphate buffer (pH = 7.0) and subsequently homogenized. Following this, the samples were centrifuged at 4°C and 12,000 revolutions per minute for 20 minutes, after which the supernatant was carefully extracted. The enzymatic reaction system was assembled using the methodology previously detailed in references (33, 34). The reaction mixture was then incubated in a 25°C water bath for 15 minutes, and finally, the optical density at 405 nm (OD_405_) was determined.

### 2.8 Histological analysis

Samples were collected three days after *B. velezensis* infection and rinsed with sterile water to remove any surface debris. Larvae from different groups were pre-fixed overnight at 4°C with a liquid fixative. The samples were then dehydrated, embedded in wax, sectioned, and stained with hematoxylin-eosin. The sections were visualized under a microscope and photographed.

### 2.9 Statistical analysis

Statistical analyses were performed using SPSS Statistics version 20 and GraphPad Prism 8.0.2 software. All data were presented as the mean ± standard deviation (SD). To evaluate the effects of different treatments on the body weight and length of housefly larvae, a two - way analysis of variance (ANOVA) was conducted, followed by Šidák correction for multiple comparisons to control the family - wise error rate.

For the antagonism experiment, data were analyzed using Student’s t - test to determine significant differences between groups. Likewise, the t - test was applied to analyze the activity of phenoloxidase in the hemolymph of the larvae. Statistical significance was indicated by asterisks, where *P < 0.05, **P < 0.01, ***P < 0.001, and ****P < 0.0001.

## 3 Results

### 3.1 Sampling, isolation, and insecticidal potential of bacteria

**Figure 1:**
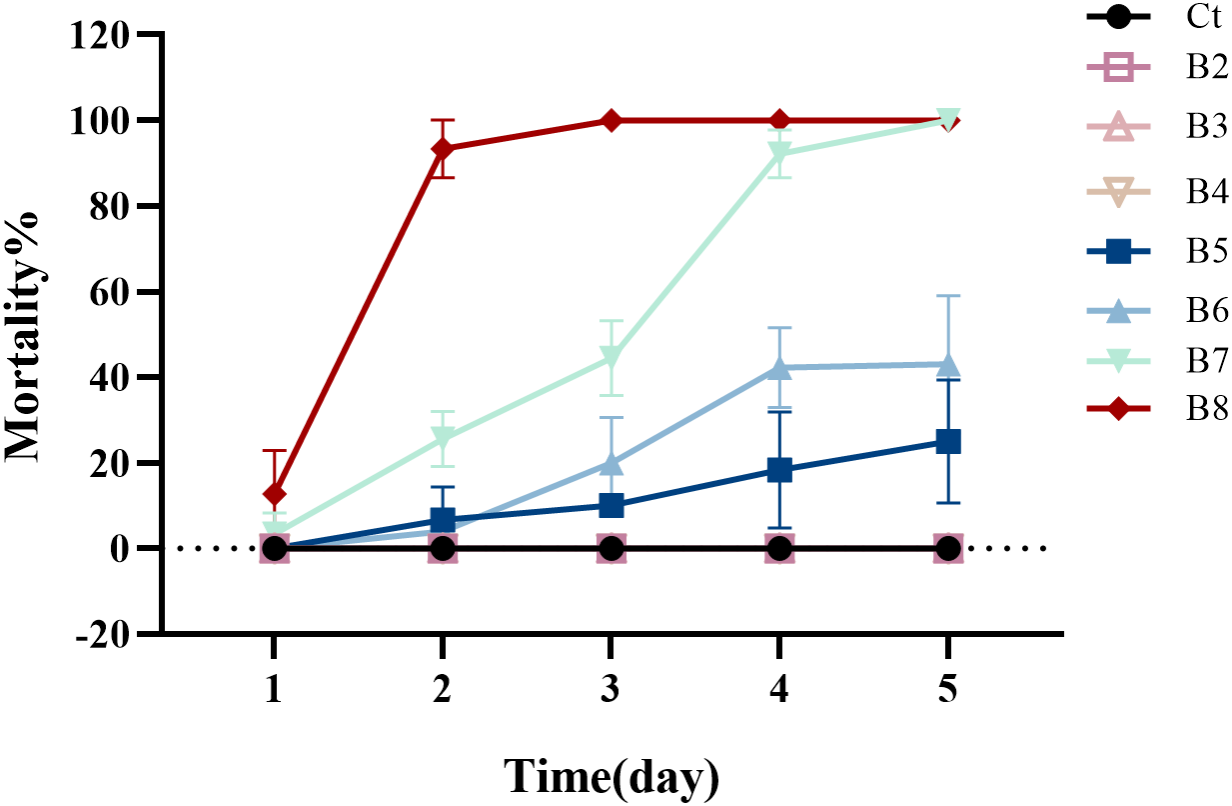
The mortality of housefly larvae following treatment with varying concentrations of *B*. *velezensis* over a 1- to 5-day period. Ct, B2, B3, B4, B5, B6, B7 and B8 represent housefly larval samples treated with LB and LB containing 10^2^, 10^3^, 10^4^, 10^5^, 10^6^,10^7^ and 10^8^ CFU/mL *B*. *velezensis*, respectively.

*B. velezensis* Bv was successfully isolated from the intestines of adult houseflies. The toxicity of the *B. velezensis* strain against 1^st^- instar housefly larvae is shown in **Figure 1**. As indicated, the toxicity of *B. velezensis* against 1^st^- instar larvae increased with higher concentrations of the bacterial solution, ranging from 2 × 10^2^ to 2 × 10^8^ CFU/mL. Larval mortality began on the first day, with relatively low toxicity observed at a concentration of 2 × 10^5^ CFU/ml, and relatively high toxicity at 2 × 10^8^ CFU/ml, where the mortality rate exceeded 90% within 48 hours.

Based on the average mortality of housefly larvae at different *B. velezensis* concentrations, the toxicity regression equation was determined to be *Y* = −5.881 + 0.915*x*. The Pearson goodness-of-fit test yielded a significance level of 0.829 (P > 0.15) (**Supplementary Tables S1, S2**), indicating that the equation is a good fit for the data.

**Supplementary Table S3** displays the concentration and 95% confidence interval of *B. velezensis* for mortality rates ranging from 1% to 99%, as analyzed by SPSS. According to **Supplementary Table S3**, the concentration at which the median lethal dose (probit) is 0.50 (LC_50_) is 10^6^ CFU/mL, with a 95% confidence limit of 10^6^ to 10^7^ CFU/mL. This information provides a theoretical basis for selecting the appropriate concentration of *B. velezensis* for controlling houseflies.

### 3.2 Genome characteristics of *B. velezensis*

Genomic information provided critical insight into the pathogenic mechanisms of *B. velezensis*. Therefore, whole genome analysis was conducted to identify the complete set of genes involved in insect lethality. The sequencing data have been uploaded to NCBI (GenBank: PRJNA1175849). The raw sequence reads of the *B. velezensis* strain were subjected to quality control, evaluation, and assembly. The strain’s whole genome consisted of a circular chromosome (**Supplementary Figure S1**) with a sequence length of 3,974,678 bp, an average G + C content of 46.35%, and an estimated size of 3.975 Mb. The genome encoded 3,983 genes, which accounted for 89.79% of the genome. The total length of the coding genes was 3,568,866 bp, with an average gene length of 896 bp. A total of 3,782 CDS were predicted in the chromosome, with a combined length of 3,508,356 (**Table 1**). The genome of strain Bv also predicted eighty-six tRNA structures, nine 23S rRNA structures, nine 16S rRNA structures, nine 5S rRNA structures, one tmRNA structures, and eighty-seven misc_RNA structures (**Table 1**). To obtain comprehensive gene function information, we performed gene function annotation using eight major databases, including UniProt, KEGG, GO, Pfam, COG, TIGERfams, RefSeq, and NR (**Supplementary Table S4**). Furthermore, alignment with the VFDB predicted that 1,161 coding sequences in the genome of the test strain could be potential virulence genes. These predicted virulence genes were classified into 13 major categories, including adherence, invasion, effect delivery system, motility, exotoxin, exoenzyme, immune modulation, biofilm, nutritional/metabolic factor, stress survival, post translational modification, anti-microbial activity/competitive advance, regulation, and information on specific virulence genes is provided in **Table 2**. Among them, we identified several important virulence genes, such as *ces*, *mucp*, and *hlyIII*.

**Table 1:** Basic information on the *B. velezensis* Bv genome.

Type	Number	Total_len	Average_len	Percentage of Genome(%)
Gene	3983	3568866	896	89.79
CDS	3782	3508356	928	88.27
tRNA	86	6665	78	0.17
23S rRNA	9	26334	2926	0.66
16S rRNA	9	13923	1547	0.35
5S rRNA	9	999	111	0.03
tmRNA	1	360	360	0.01
misc_rna	87	12229	141	0.31

**Table 2:** Predictive classification statistics of virulence factors of strain BV.

Primary Classification	Number of Genes	Secondary Classification (Number of Genes)
Defensive virulence factors	391	Immune modulation (325), Stress protein (24),biofilm (34), antimicrobial activity(8)
Offensive virulence factors	336	Adherence (87), toxin (114), secretion system (32), invasion (12), motility (91)
Nonspecific virulence factor	12	Iron uptake system (4), exoenzyme (8)
Regulation of virulence-associated genes	400	Regulation (67), post translational modification (7), nutritional/metabolic factor(326)

### 3.3 The impact of *B. velezensis* proliferation on the growth and development of housefly larvae

**Figure 2:**
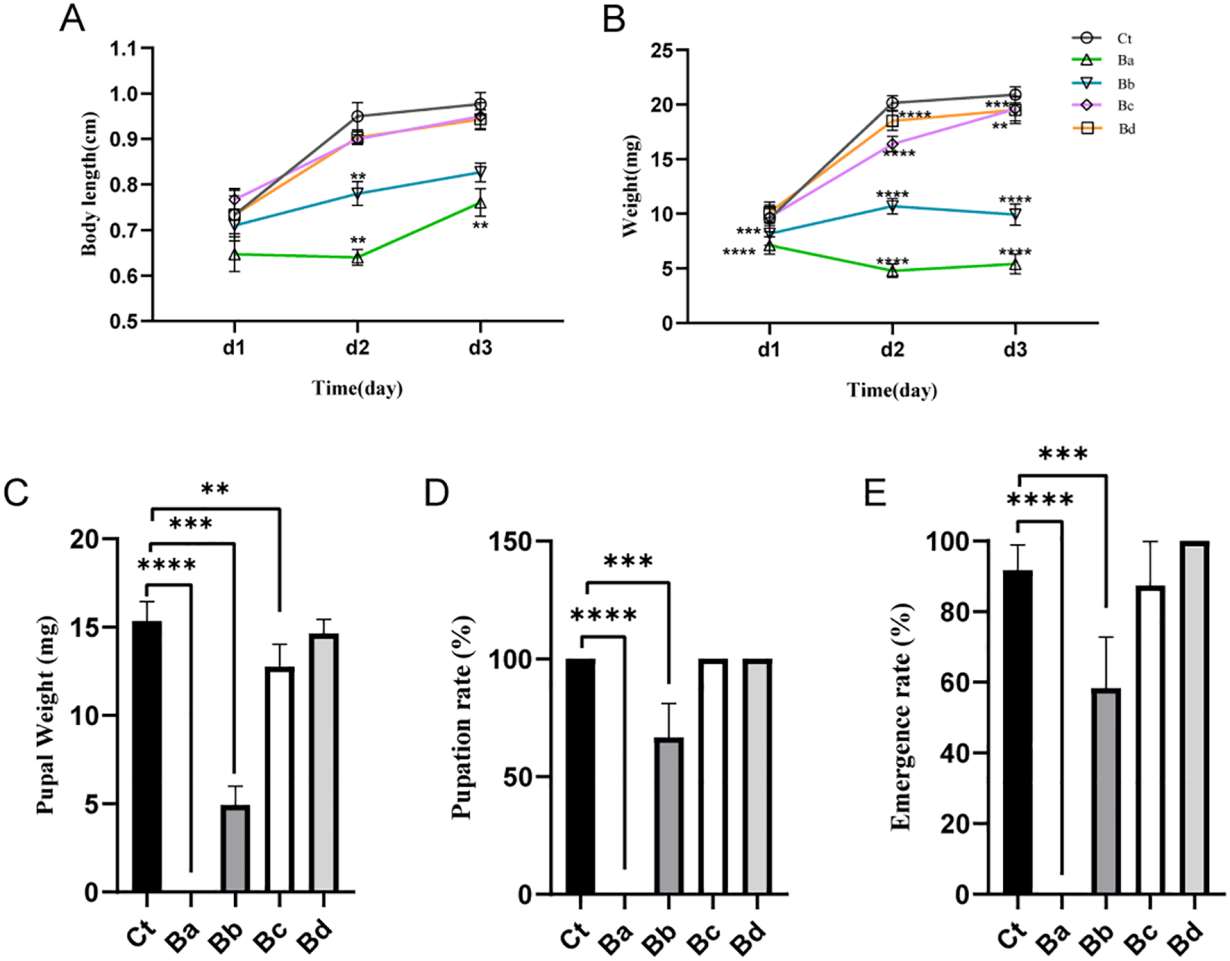
Developmental changes in housefly larvae subjected to different treatments. **(A)** Significant changes in the body weights of housefly larvae over time across different treatments. **(B)** Significant changes in the body lengths of housefly larvae over time across different treatments. The effects of various treatments on **(C)** pupal weight, **(D)** pupation rate, and **(E)** emergence rate of the housefly. Ct, Ba, Bb, Bc, and Bd represent housefly larval samples treated with LB and LB containing 108, 106, 104, and 102 CFU/mL B. velezensis, respectively. Data are presented as means ± SEMs. Repeated measures ANOVA followed by Sidak correction for multiple comparisons was used. **P < 0.01, ***P < 0.001, ****P < 0.0001; n.s., not significant.

Various diluted concentrations of *B. velezensis* were incorporated into the diet of housefly larvae, and the effects on larval body weight, length, pupal weight, pupation rate, and emergence rate were analyzed under different dietary conditions. Compared with the control group, larvae fed with high concentrations of *B. velezensis* (Ba and Bb) showed almost no growth, with reduced body length and body weight being only 1/4 and 1/2 of that of the control larvae on days 2-3. As the concentration of *B. velezensis* in the larval diet decreased, its inhibitory effect on the growth of housefly larvae also decreased slightly. In the Bc and Bd groups, there was no difference in body weight compared to the control group on the first day, but a 10% to 25% decrease in body weight was observed on the second and third days (**Figure 2**). Similarly, the development of housefly larvae fed with *B. velezensis* was significantly inhibited, with high concentrations leading to larval death and failure to form normal pupae (**Figure 2**).

### 3.4 Histological analysis

**Figure 3:**
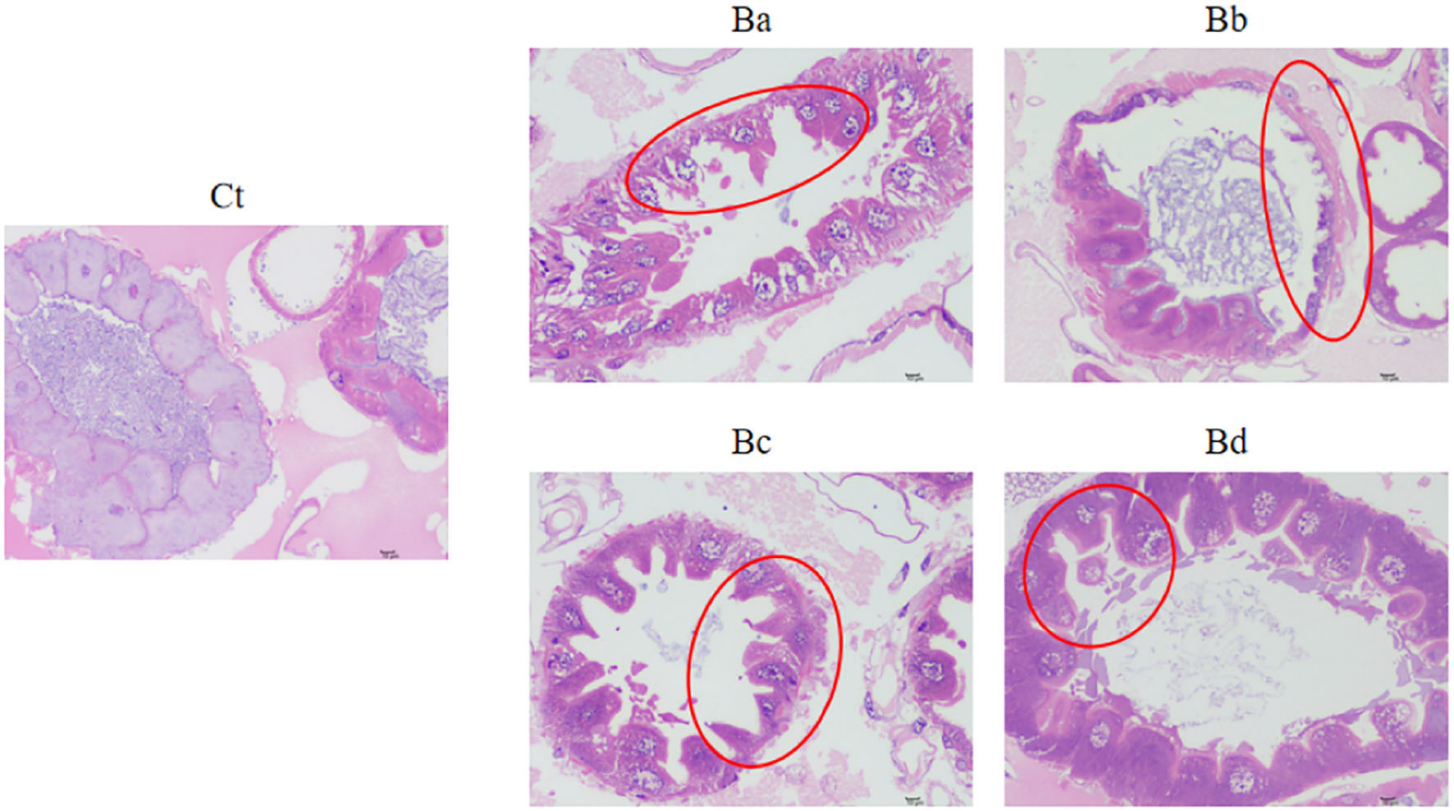
Intestinal damage in housefly larvae. Representative H&E-stained images of larvae collected from the Ct and Ba-Bd group. Ct, Ba, Bb, Bc, and Bd represent housefly larval samples treated with LB and LB containing 10^8^, 10^6^, 10^4^, and 10^2^ CFU/mL *B*. *velezensis*, respectively.

To further investigate the impact of infection on gut morphology, histological analyses were performed. **Figure 3** shows the histological changes observed in the guts of larvae exposed to *B. velezensis*. Compared to the control group fed with water, the gut cells of the infected larvae exhibited degeneration and shrinkage, with condensed nuclei and reduced cytoplasmic content. Additionally, the epithelial cells of the infected larval contained numerous vacuoles. These modifications were even more pronounced at high concentrations of *B. velezensis* (**Figure 3**).

### 3.5 Comparative analysis of intestinal microbiota in different groups of housefly larvae

Gut bacteria have significant effects on the growth and development of housefly larvae. We analyzed the fluctuations in gut microbiota in housefly larvae treated with *B. velezensis* using 16S rRNA (BioProject ID: PRJNA1175836).

**Figure 4:**
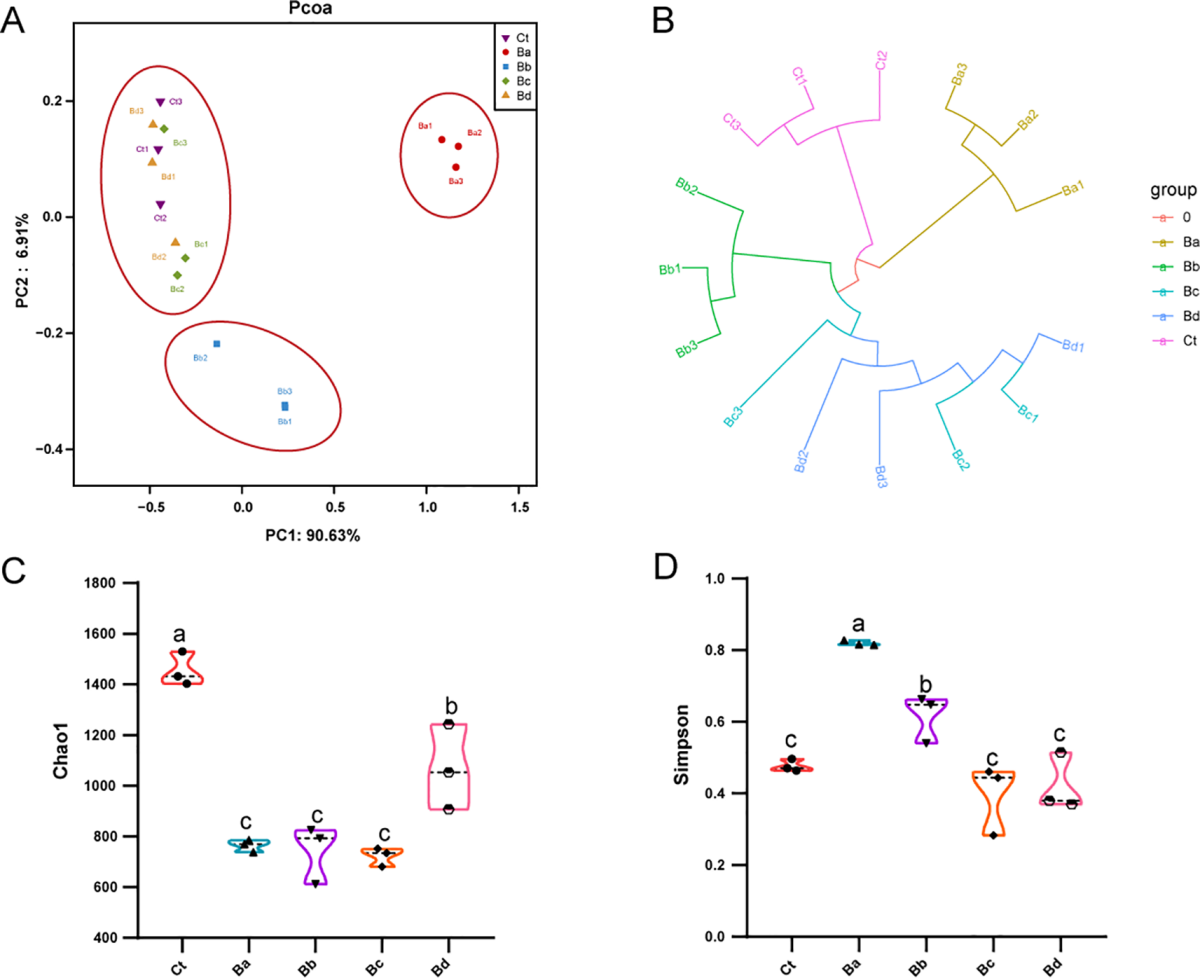
**(A)** PCoA of bacterial community structures in the different groups, with each symbol representing one sample of intestinal bacteria. **(B)** UPGMA tree analysis of sample evolution. **(C)** Chao1 and **(D)** Simpson indices of intestinal bacteria in the different housefly groups. Ct, Ba, Bb, Bc, and Bd represent housefly larval samples treated with LB and LB containing 108, 106, 104, and 102 CFU/mL B. velezensis, respectively. Data are presented as mean ± SEM. Multiple comparisons were conducted using a Tukey test.

**Figure 5:**
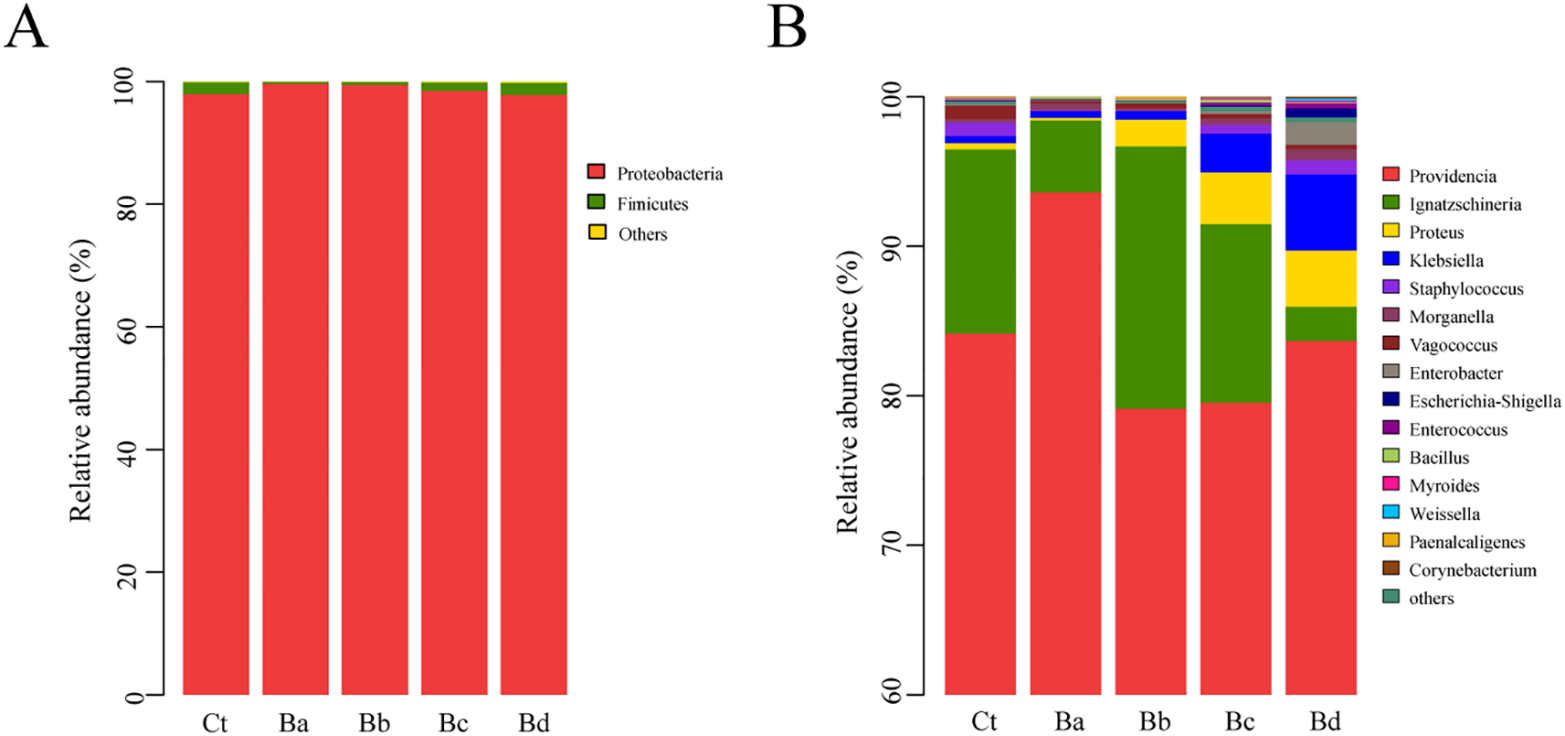
Relative abundances and distributions of the dominant bacterial **(A)** phyla and the top 15 bacterial **(B)** genera in housefly larval samples on the 3^rd^ day. Bacterial genera with abundances over 1% in at least one sample were classified as major genera, while those with percentages lower than 1% in all samples were classified as minor genera.

PCoA revealed low similarity in microbial community structures between the control group and the treatment group. Additionally, the Chao1 and Simpson indices indicated significant changes in bacterial diversity and richness, particularly in the Ba group (P < 0.05; **Figure 4**). No significant differences were observed in the proportions of each taxon at the phylum level. At the genus level, 15 primary bacterial taxa were identified, including *Providencia*, *Ignatzschineria*, *Proteus*, *Staphylococcus*, *Morganella*, *Vagococcus*, and *Enterobacter*, among others (**Figure 5**). Moreover, housefly larvae treated with high concentrations of Bv exhibited more pronounced changes in bacterial composition at the genus level. Compared to the Ct group, the content of *Providencia* and *Morganella* increased significantly in all treatment groups (P < 0.05), with notable increases of 9.43% and 0.24%, respectively, in the Ba group. Conversely, the content of *Enterobacter* decreased in the intestinal contents of the *B. velezensis*-treated groups, with a reduction of 0.07% (**Figure 5**).

### 3.6 Mutual benefits and competition between *B. velezensis* and cultivable bacteria in the housefly intestine

**Figure 6:**
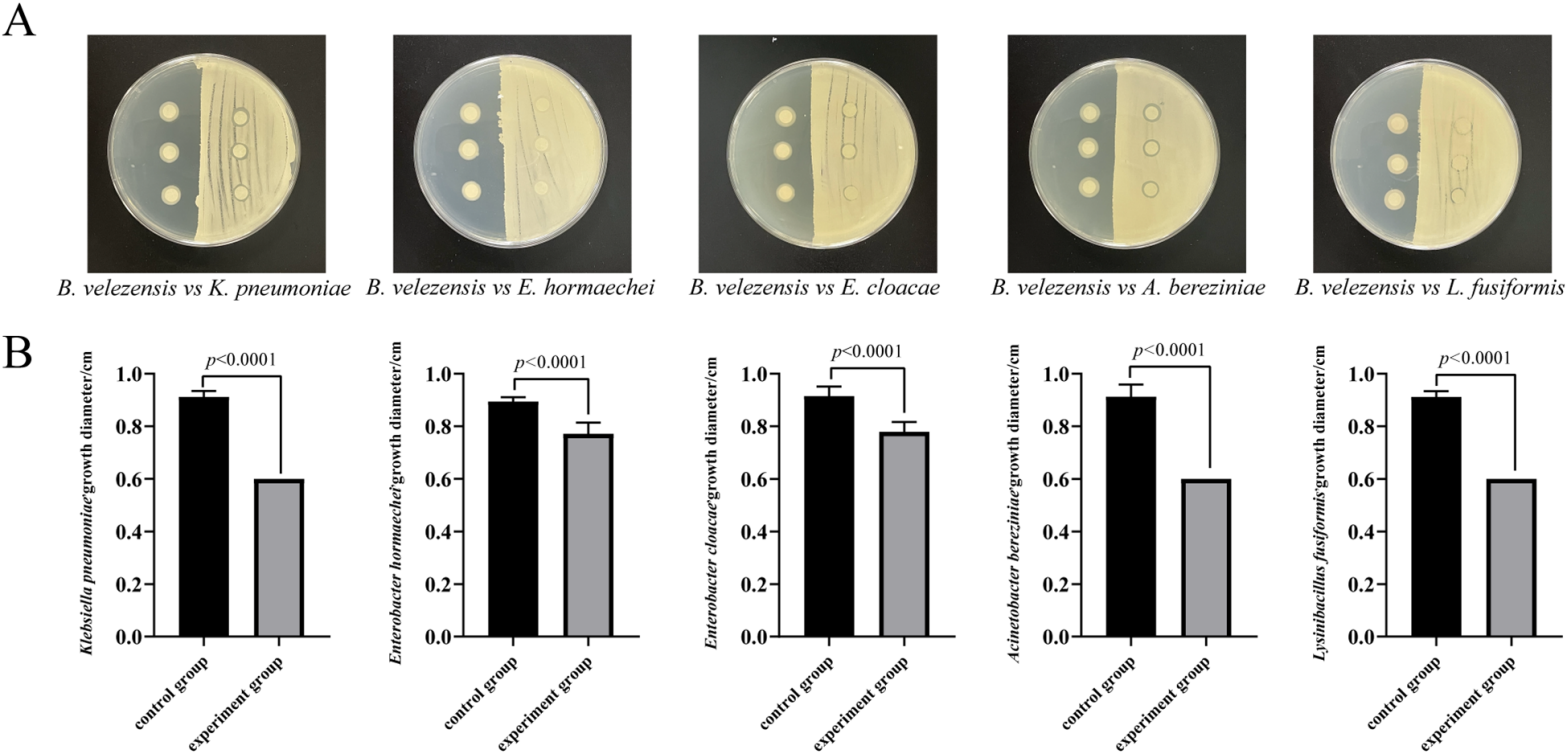
Antagonism experiment between *B*. *velezensis* and *K*. *pneumoniae*, *E*. *hormaechei*, *E*. *cloacae*, *A*. *berezinia*, and *L. fusiformis* in the housefly larval intestine. **(A)** *B*. *velezensis* was seeded on the right side of the plate, with sterile water spread on the opposite side as a control. Filter paper was dipped into *E*. *hormaechei*, *K*. *pneumoniae*, *E*. *cloacae*, *L. fusiformis*, and *A*. *bereziniae*. **(B)** Competitive inhibition between *B*. *velezensis* and *K*. *pneumoniae*, *E*. *hormaechei*, *E*. *cloacae*, *A*. *berezinia*, and *L. fusiformis* in the housefly larval intestine. Data are presented as mean ± SEM. Statistical analysis was performed using the t-test.

To simulate the interactions between invasive bacteria and other culturable bacteria in the larval intestine, we conducted a short-term *in vitro* bacterial culture experiment. We isolated 11 cultivable bacterial species from the intestine of housefly larvae, including *Enterobacter hormaechei*, *Klebsiella pneumoniae*, *Pseudomonas aeruginosa*, *Acinetobacter bereziniae*, *Providencia stuartii*, *Enterobacter cloacae*, *Lactococcus lactis*, *Lysinibacillus fusiformis*, *Providencia vermicola*, *Bacillus safensis*, and *Serratia marcescens*. These were used for antagonism assays with *B. velezensis.* The results indicated that *B. velezensis* inhibited the growth of *K. pneumonia*, *E. hormaechei*, *E. cloacae*, *A. berezinia*, *L. fusiformis*, and *B. safensis* (**Figure 6**). Our previous studies have reported that *E. hormaechei* and *K. pneumoniae* are beneficial bacteria in the housefly larval gut and promote larval growth, while *Providencia* and *S. marcescens* are harmful and have lethal effects on the larvae. These results help clarify the role of *B. velezensis* in the gut microbiome and its impact on larval growth and development.

### 3.7 Gene expression alterations induced by *B. velezensis* exposure

**Figure 7:**
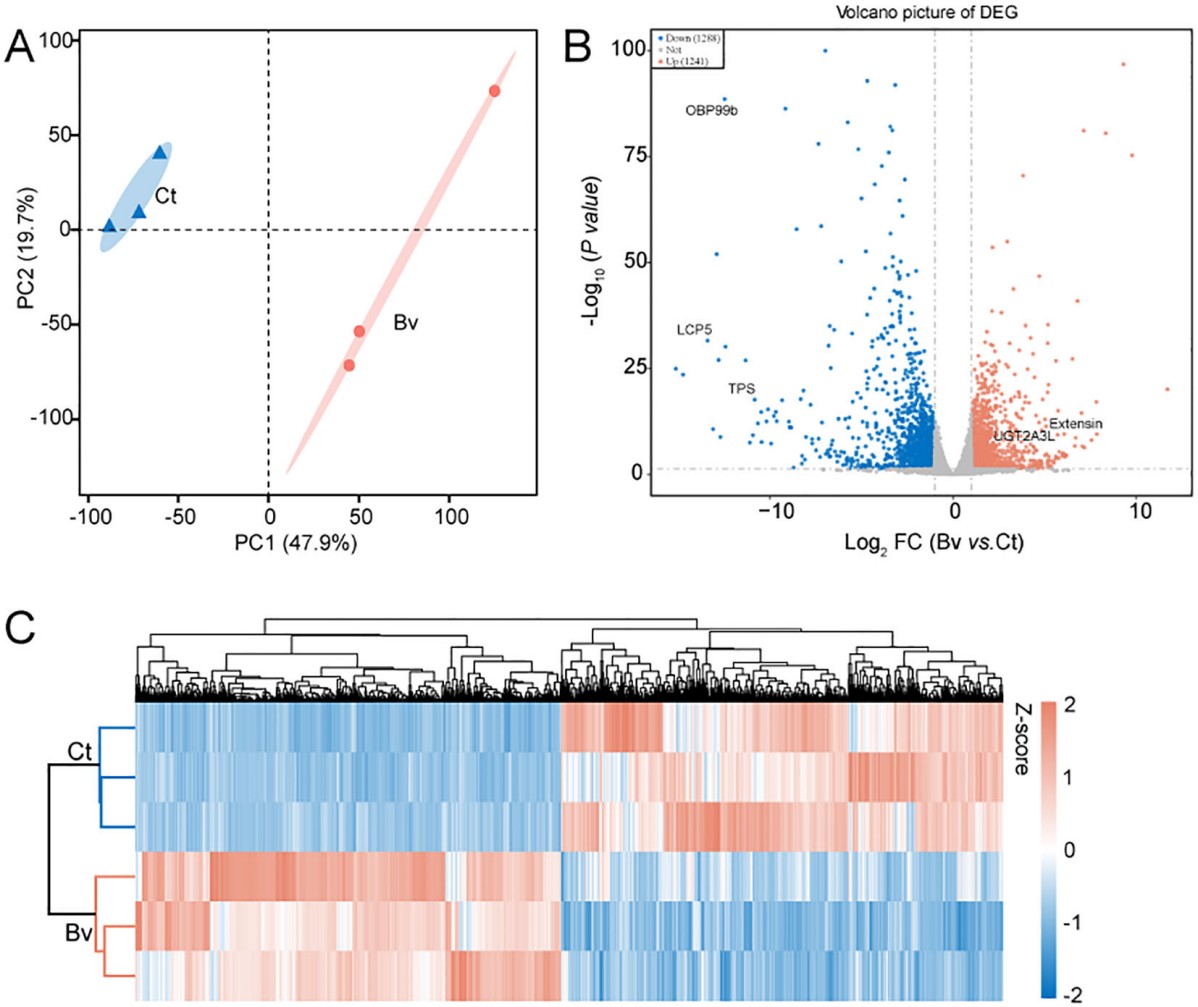
Differences in gene expression between the Ct and Bv group. **(A)** PCA illustrates clustering of *B*. *velezensis* treatments in a 2D space defined by the first two principal components (PC1 and PC2), which together accounted for 67.6% of the dataset’s variation. **(B)** Volcano plot of DGEs. The five significantly altered DGEs are OBP99b (general odorant-binding protein 99b), LCP5 (larval cuticle protein 5), TPS (alpha,alpha-trehalose-phosphate synthase), Extensin and UGT2A3L (UDP-glucuronosyltransferase 2A3-like). The horizontal axis represents the fold change in gene expression, while the vertical axis represents the statistical significance of the change. Each point represents a gene, red points indicate significantly upregulated genes, blue points indicate significantly downregulated genes, and the gray points indicate genes without significant differential expression. **(C)** Hierarchical clustering heatmap of DEGs in the Ct and Bv groups.

To examine the effects of our experimental treatments on the function and structure of larval genes at an overall level, we performed transcriptome analysis (BioProject ID: PRJNA1175847) to uncover the underlying mechanisms. Given with a q-value < 0.05 and at least a twofold change (log2(fold change) > 1 or < −1) were considered differentially expressed genes (DEGs). Principal component analysis (PCA) of the transcriptome data showed that PC1 accounted for 47.9% of the variance in gene expression and separated the Bv group from the Ct group, indicating a significant impact of *B. velezensis* on the intestinal transcriptome profile (**Figure 7A**). A comparison of DEGs in the Bv group relative to the control revealed over two thousand genes with significantly altered expression levels. Specifically, 1241 genes were upregulated, and 1288 genes were downregulated (**Figure 7B**). Notably, the expression levels of genes such as UDP-glucuronosyltransferase (LOC101893291) and extensin (LOC101894124) were greatly increased (>100-fold) in the Bv group. In contrast, larval cuticle protein 5 (LOC101900538), general odorant-binding protein 99b (LOC101887971) and alpha,alpha-trehalose-phosphate synthase [UDP-forming] (*TPS*) was significantly reduced (>100-fold) in the Bv group.

**Figure 8:**
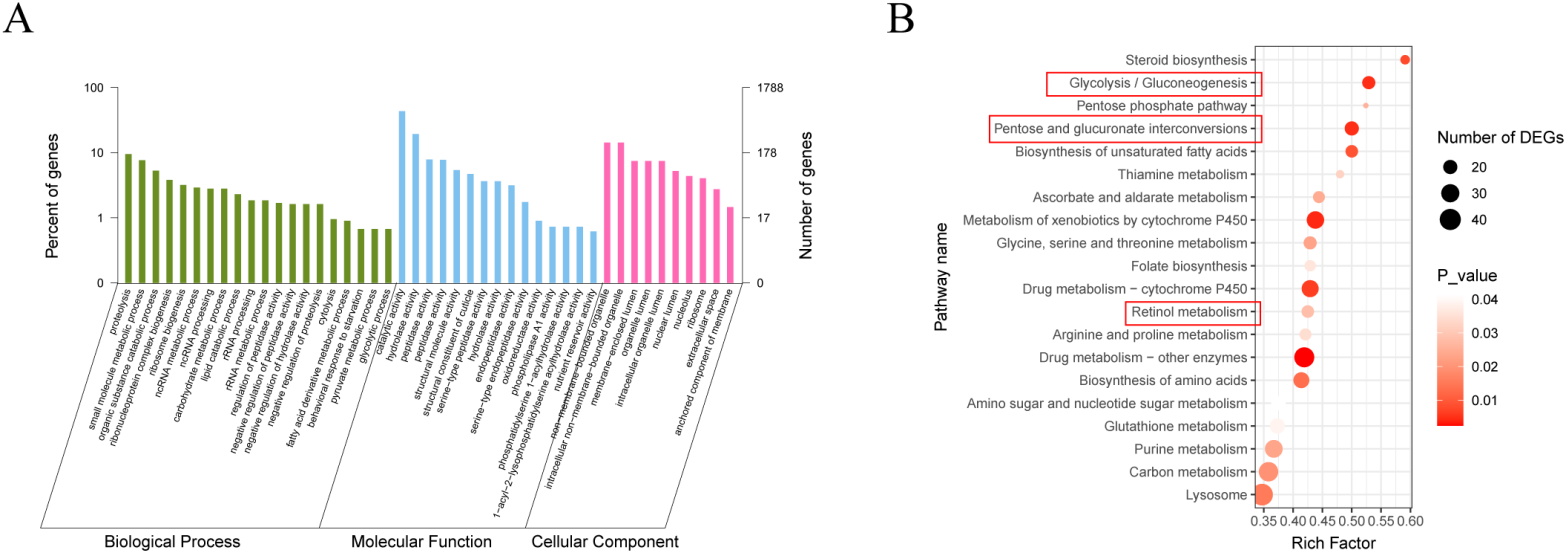
GO and KEGG analysis of differentially expressed genes in the gut of larvae after feeding with *B*. *velezensis.* **(A)** GO enrichment analysis of DEGs (Q value < 0.05). DEGs were categorized into three GO categories: biological process, cellular component, and molecular function. **(B)** KEGG enrichment analysis of DEGs (Q value < 0.05). Statistics show the top 20 enriched pathways of differentially expressed genes in each pairwise comparison. A higher rich factor indicates greater enrichment. The Q value is corrected p-value ranging from 0 to 1, with lower Q-values indicating higher enrichment.

The expression heatmap of DEGs between the control and the Bv groups, shown in **Figure 7C**, further illustrates the significant differences in gene expression patterns between the two groups. To better understand the functions of DEGs and their related pathways, GO enrichment and KEGG analysis were conducted (**Figure 8**). The enrichment analysis revealed that 20 biological processes were over-represented, along with 15 molecular functions and 10 cellular components (**Figure 8A**). The GO terms with the highest number of affected transcripts included “proteolysis” (169 DEGs), “small molecule metabolic process” (136 DEGs), “catalytic activity” (770 DEGs), “hydrolase activity” (345 DEGs), “non−membrane−bounded organelle” (255 DEGs), and “intracellular non−membrane−bounded organelle” (255 DEGs). KEGG pathway enrichment analysis identified 20 significantly affected pathways (**Figure 8B**), including retinol metabolism, glycolysis/gluconeogenesis, and pentose and glucuronate interconversions. Notably, genes enriched in the glycolysis/gluconeogenesis pathway were significantly downregulated, while genes in the retinol metabolism pathway were significantly upregulated.

### 3.8 Effects of feeding *B. velezensis* on phenoloxidase activity in housefly larvae

**Figure 9:**
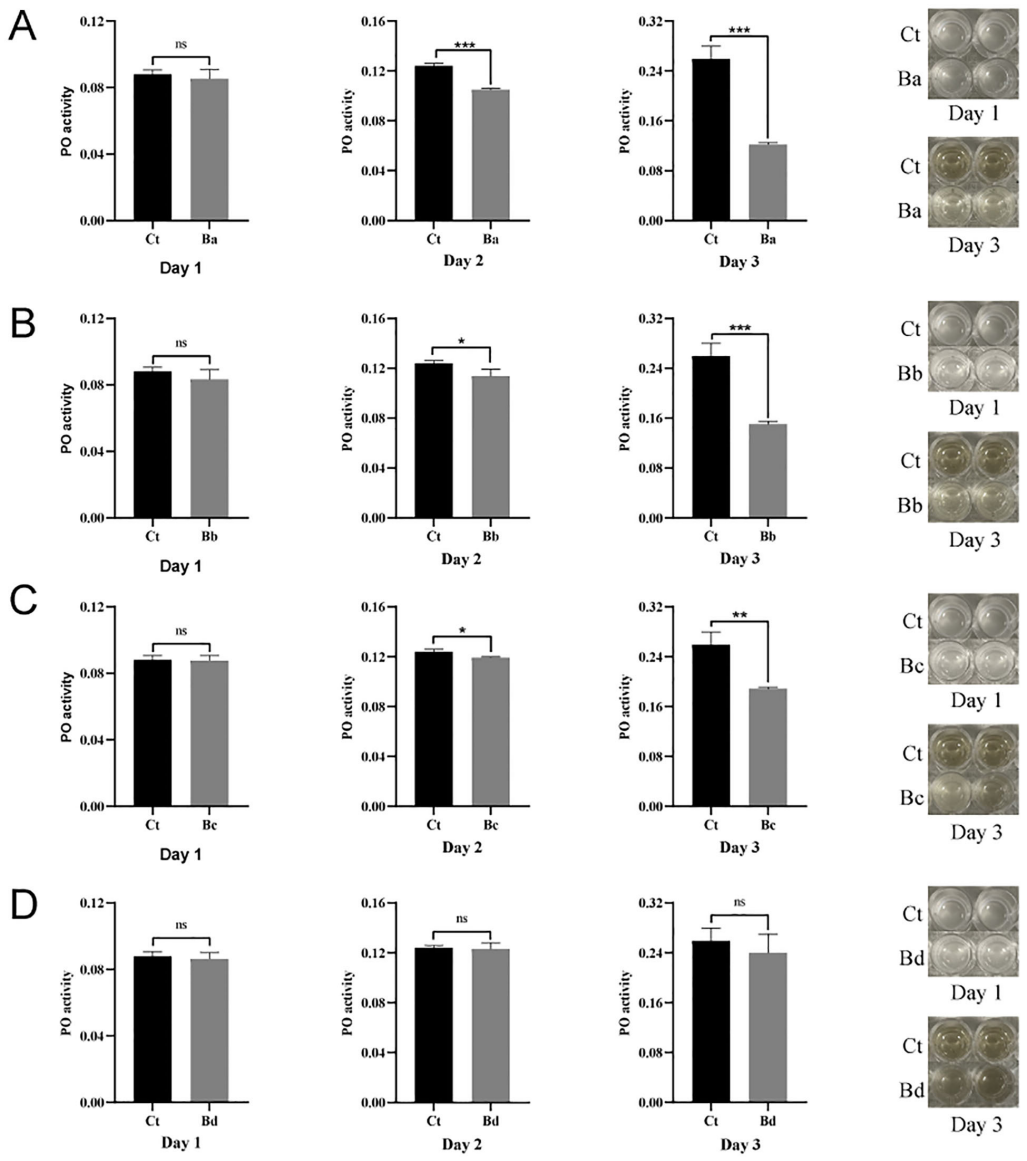
Effects of *B. velezensis* in the guts of housefly larvae on phenoloxidase activity in the hemolymph. Days 1–3 represent the developmental period of housefly larvae. Data are presented as mean ± SEM. Statistical analysis was performed using the t-test. *P < 0.05, **P < 0.01, ***P < 0.001; n.s., not significant.

The mechanism of *Bacillus* toxicity may be associated with a reduced immune response in the host (35–38). To investigate this, we analyzed the impact of *B. velezensis* on phenoloxidase (PO) activity and the PO cascade-mediated melanization, which are key components of innate immunity in larvae. The results indicated that, on the first day after feeding *B. velezensis* to housefly larvae, there were no difference in phenoloxidase activity or melanization ability in the hemolymph among the groups (P>0.05). However, on the 2^nd^ and 3^rd^ day, the larvae in the Ba, Bb, Bc groups exhibited no melanization and significantly inhibited phenoloxidase activity compared to the control group, with the more pronounced effects observed on the 3^rd^ day. In contrast, larvae fed housefly larvae the minimum concentration of bacteria (Bd) showed no significant difference in phenoloxidase activity in the hemolymph over the three days. Additionally, the results demonstrated that phenoloxidase activity in the hemolymph of housefly larvae increased progressively with the larvae development (**Figure 9**).

## 4 Discussion

*Bacillus* spp. are widely distributed and exhibit strong resistance to external harmful factors. Most species within the genus are beneficial microorganisms. Some *Bacillus* species are increasingly used in various applications, including anti-insect fungicides, surface-active agents, biological agents, flavor enhancers, and nutritional and healthcare products (39). Among these, *B. velezensis*, a relatively new species of *Bacillus*, has shown promise in promoting plant growth and inhibiting plant pathogens, making it a potential in biocontrol agent in agriculture (40). Additionally, *B. velezensis* can be utilized in industry to degrade industrial harmful by-products and has broad applications in biomedicine. Despite this, research on the insecticidal activity of *B. velezensis* is limited. The effects of *B. velezensis* on the insect immune systems and gut microbiota during infection remains unclear. In this study, we confirmed that *B. velezensis* effectively eradicates flies. We identified that *B. velezensis* inhibits the humoral immune response of housefly larvae by reducing phenoloxidase activity and disrupts their growth and development by altering gut microbiota.

### 4.1 Analysis of bacterial genomics

*B. velezensis* was previously reported to eradicate *Aedes aegypti* (Diptera: Culicidae) larvae with low toxicity to non-target species (41). Our study investigated whether *B. velezensis* derived from houseflies exhibits fly-killing activity. Based on observations from toxicity tests and data analysis, higher concentrations of *B. velezensis* were found to be more effective in killing housefly larvae. To explore the key mechanisms underlying Bv insecticidal activity, we characterized the genomes of the strains selected in this study. Genomic annotation revealed multiple bacterial virulence factors in *Bacillus*. The *ces* gene is a toxin gene that causes vomiting. The *Bacillus cereus*, the *ces* gene can be transcribed and translated into enterotoxins, leading to vomiting-type food poisoning and even sepsis (42–46). Hemolysin III (*hlyIII*) and enterotoxins (*entCW/entB*) are hemolytic toxins of *Bacillus* (47, 48). Hemolysins are known for their cytotoxic and hemolytic activities, anti-microbial properties, and various degradative enzymes, which may enable the bacteria to thrive in the intestinal environment and damage the intestinal barrier (49). Metalloproteinases are also virulence factors in *Bacillus* and may play a role in their pathogenic processes. Studies have reported that *P. luminescens* secretes two virulence factors abundantly, the gut-active toxin complex A (*Tca*) and the metalloprotease PrtA, which may facilitate the rapid destruction of gut tissue (50); Metalloproteinase A2 contributes to Bt virulence by aiding the bacterium in crossing the gut barrier into the haemocoel (51). Members of the Immune Inhibitor A metalloprotease family, including *inhA2* and *inhA3*, help bacteria resist insect immune defenses by degrading AMPs (Antimicrobial Peptides) and hydrolyzing various proteins and cellular components (e.g., *fibronectin*, *collagens*, and *laminin*) (52–54). In addition, a 2021 study by Liang and colleagues identified and characterized a novel *B. velezensis* strain, ATR2. This strain demonstrated remarkable antibacterial, antifungal, and insecticidal activities across a wide spectrum. Genome analysis showed that *B. velezensis* ATR2 possessed a great capacity for synthesizing kinds of secondary metabolites, which are associated with the biocontrol of multiple plant diseases (55–57). Moreover, the extracellular metabolites from *B. velezensis* ATR2 were also proven to be efficacious in control of aphids in laboratory bioassays and in the field, where corrected mortalities of multiple aphids were almost up to 90% (58). Therefore, we speculate that *B. velezensis* may secrete many bioactive compounds with insecticidal, cytotoxic, and anti-microbial activities, which ultimately exert an impact on the growth and development of the host. However, further studies are warranted to validate this hypothesis.

### 4.2 Gut microbiota analysis

Insects host a diverse array of microorganisms in their guts, including bacteria, fungi, protists, and archaea (59). These microorganisms provide significant physiological and ecological advantages to their hosts (19), playing a crucial role in insect development, nutrition, immunity, metabolism, and resistance to pathogen colonization (17, 60–65). Our observations revealed that the invasion of pathogenic-like bacteria, such as *B. velezensis*, altered the composition of the host gut microbiota. This finding aligns with previous research indicating that disruptions in gut microbiota homeostasis are linked to suppressed growth and development in housefly larvae. Although previous studies have explored the *B. velezensis* as a probiotic to modulate microbiota composition of certain animals (66–68), their impact on the gut microbiota of insects have rarely been explored. In our study, *B. velezensis* was identified as a potential insect pathogen, feeding *B. velezensis* had a notable impact on the commensal bacteria of housefly larvae at the genus level. Notably, we observed an overgrowth of *Providencia*, a bacterium known for its high-mortality rate in flies (69). Research indicates that *P. stuartii* and *P. vermicola*, as “harmful bacteria” in housefly larvae, can significantly inhibit their growth and development (34), highlighting the substantial role of *Providencia* in housefly mortality. Conversely, our findings revealed a decrease in the *Enterobacteria* population within the gut of housefly larvae. Previous studies have documented that ingestion of the probiotic strain *E. hormaechei* by housefly larvae can enhance their growth, accelerate developmental processes, and boost humoral immunity (34, 70). Moreover, research under axenic conditions demonstrated that inoculation with *E. cloacae* confers increased resistance to infection, thereby improving the overall fitness of the larvae (71). Similar to the results of the present study, a newly identified bacterial strain, *Bacillus velezensis* LP16S, was shown to modify the internal microbiota of the southern green stink bug (*Nezara viridula*). This alteration in microbiota composition significantly impacts the survival rate and lifespan of *N. viridula*, highlighting *B. velezensis* LP16S as a potential entomopathogen for the effective management of this agricultural pest (72). Furthermore, *B. thuringiensis* infection can alter the abundance and structural composition of the intestinal bacteria community in the lepidopteran pest *Spodoptera exigua*, with dysbiosis of the gut microbiota significantly affecting Bt pathogenicity (73, 74). Guillaume Tetreau et al. (75) found that mosquito larval bacterial microbiota was strongly affected by *B. thuringiensis israelensis* (Bti) infection after only a few hours of exposure (75). Wang et al. (76) showed that treatment with Bt05041 significantly enriched the gut bacterial community, altered the composition of the gut microbiota, and resulted in the dysfunction of gut cells (76). Meanwhile, several studies have been reported that the interaction between Bt and gut microbiota can be competitive. *B. thuringiensis* can produce bacteriocin to inhibit the growth of gut bacteria. Thus, it is plausible that *B. velezensis* induces the overgrowth of certain pathogenic strains while suppressing beneficial bacteria through niche competition and nutrient limitation. To further investigate this competition mechanism, we conducted a plate antagonism experiment. The results demonstrated that *B. velezensis* significantly inhibited the growth of *K. pneumoniae*, *E. hormaechei*, *E. cloacae, A. berezinia*, and *L. fusiformis.* Consequently, the invasion of *B. velezensis* altered the interactions among gut microbial communities and accelerated the mortality of housefly larvae.

There is study report that normal gut microbiota mediated pathogen clearance from the gut lumen (77); this suggests that the gut microbiota can act as another form of protection response in organisms, or at least an important complement to host gut immune protection (73). Disruption of the gut tissue facilitates the indigenous gut microbiota to access the hemolymph and other part of the tissues. Researchers of the past decades advocating that after the midgut damage caused by the Bt toxins, the midgut epithelium allows the gut microbiota access to the hemolymph and other parts of the larval body, where they switch from commensal to pathogen and eventually lead to fatal septicemia (78–81). Although we did not evaluate microbes in the hemolymph, we found that the damage of the intestinal epithelium of housefly larvae was discovered by H&E-stained. Therefore, dysbiosis of the gut microbiota of insects caused by *B. velezensis*, can increase the probability of opportunistic pathogens and further result in insect death.

### 4.3 Transcriptomic analysis

The gut microbiota not only provides essential nutrients to the host (82, 83) but also aids in insect resistance to environmental stress by regulating host-signaling pathways (84, 85). The main metabolic functions of the gut bacterial communities included amino acid transport and metabolism, carbohydrate transport and metabolism, inorganicion transport and metabolism, vitamin biosynthesis, lipid digestion, energy metabolism, and protein digestion (86, 87). However, by employing transcriptomic approaches, including KEGG and GO, we found that 10 of the 20 KEGG pathways identified in the gut tissue of housefly larvae related to metabolism, mainly the metabolism of thiamine, vitamins, amino acids, energy, retinol. These results suggest that *B. velezensis* treatment induced distinct alterations in gut microbial functions related to cellular metabolism. We identified the pentose and glucuronate interconversions pathway as the primary abnormal metabolic pathway, which is closely linked to the liver’s detoxification ability (88) and may contribute to disease progression. Moreover, we observed that retinol metabolism was activated in houseflies following *B. velezensis* exposure. Upon activation of retinol metabolism, an increased amount of retinol is converted to retinoic acid (89, 90). In our previous study (91), feeding experiments demonstrated while retinol supports the development of housefly larvae, retinoic acid does not. It is speculated that *B. velezensis* activates retinol metabolism, promoting the conversion of retinol to retinoic acid, which in turn mediates larval growth retardation. Additionally, we observed that glycolysis/gluconeogenesis was inhibited following the introduction of *B. velezensis*. The findings are consistent with previous studies. For example, 3-bromopyruvate (3-BrPA), a typical glycolytic inhibitor, has been shown to inhibit glycolysis, disrupts carbohydrate homeostasis, and ultimately arrest the growth and development of *Hyphantria cunea* larvae. Furthermore, research indicates that interference with carbohydrate metabolism directly or indirectly affects insect chitin synthesis (92–94). Chitin, predominantly found in the exoskeleton and the peritrophic matrix (PM) of insects, plays a crucial role in the growth, development, and metamorphosis (95). Given the critical functions of glycolysis in regulating carbohydrate metabolism and ATP generation (96–100), we believe that the growth retardation of housefly larvae fed with *B. velezensis* is likely due to an obstruction of anabolism and a shortage of ATP caused by *B. velezensis*-induced glycolysis inhibition.

Differentially expressed gene analysis was conducted to uncover the molecular mechanisms linking bacterial invasion to insect development. The results revealed that *B. velezensis* upregulated genes coding for UDP-glucuronosyltransferase (LOC101893291) and extensin (LOC101894124). Konno et al. suggest that proteins with an extensin domain may function as swelling or gel-forming agents. Specifically, the MLX56 family proteins can use their extensin domains (which have a gum arabic-like structure), as swelling agents, expanding the peritrophic membrane (PM), a thin membrane composed of chitin and protein in the insect midgut lumen, into an abnormally thick membrane that inhibits insect growth (101). Additionally, we observed the upregulation of UDP-glucuronosyltransferase (LOC101893291), consistent with our previous studies; its increased expression may contribute to the activation of xenobiotics metabolism and elevate retinoic acid levels through autoregulatory negative-feedback loops (91). Furthermore, the larval cuticle protein 5 (LOC101900538) was significantly downregulated in the Bv group. Cuticular proteins (CPs) are critical components of the insect cuticle and play essential roles in maintaining normal insect morphology, development, and defense against various external environmental stresses (102, 103). Research shows that dysfunctional CP genes, such as *BdCPAP3* genes in *B. dorsalis*, can lead to developmental defects by altering normal larval cuticle properties, such as reducing chitin content and disrupting chitin arrangement (104). We also found that a gene related to odorant-binding proteins (OBPs) (LOC101887971) was downregulated. OBPs, as specific chemosensory proteins, are potentially involved in anti-inflammatory actions, regeneration, development, and reduced insecticide susceptibility (105, 106). Recent studies have reported that silencing chemosensory genes increases insect susceptibility and causes high mortality upon insecticide exposure (107, 108). For example, odorant-binding protein 2 reduces imidacloprid susceptibility in *Diaphorina citri* (106); while efficient silencing of TcasOBPC01 increases larval susceptibility to insecticides and causes higher mortality in *T. castaneum* (108). These findings indicate that chemosensory genes play vital roles in insect chemical defense mechanisms (106). Meanwhile, we observed a significant reduction in *TPS* genes, which an crucial for energy production, growth and development, metamorphosis, stress recovery, chitin synthesis, insect flight, and other biological processes (109–112). Chen et al. reported that *TPS* gene disruption is lethal at early larval stages in *D. melanogaster*. Knockdown of *LdTPS* delayed development, strongly reduced trehalose content, and caused larval and pupal lethality in *Leptinotarsa decemlineata* (113). In *N. lugens*, three abnormal phenotypes or death occurred when *TPS1* or *TPS2* expression was significantly reduced by RNAi, disrupting chitin metabolism balance upon *TPS* gene knockdown (114–116). In *S. exigua*, RNAi*-*induced *TPS* knockdown causes larval and pupal lethality (93). An increasing number of studies have shown significant insect mortality when the *TPS* gene is disrupted (93, 117), suggesting that *TPS* may be a suitable target for potential pest control inhibitors. Therefore, *B. velezensis* may function as a *TPS* inhibitor, making it an ideal insecticidal agent.

### 4.4 Analysis of PO activity

The innate immune system in insects generates stress responses to defend against foreign species during pathogenic microorganism invasion (118). Like other insects, houseflies lack acquired immunity and can resist various pathogens solely through their innate immune response. The prophenoloxidase-activating system in insect hemolymph, a crucial component of the innate immune defense, plays an indispensable role in immune defense (119). In this study, we investigated the phenoloxidase activity and melanization response of housefly larvae during Bv infection. The results showed that the phenoloxidase activity of housefly larvae weakened after feeding on the bacteria, which reduced the larvae’s immunity and defense capabilities. Additionally, the insect immune system not only protects the host against pathogen infection but also regulates the colonization of symbiotic microorganisms in the gut to maintain host homeostasis (120). It has been reported that the native gut microbiota of bees is associated with the upregulation of anti-microbial peptides (AMPS), such as apidaecin and hymenoptaecin (121). The induction of AMPS is a type of humoral immune response in insects (122); Futo et al. (123) reported that *Tribolium castaneum* larvae with a reduced microbiota load showed a decreased survival rate upon immune challenge by Bt (123), indicating that gut microbiota is essential for immune priming. At the LC_50_ dose of Bt, the destruction of intestinal cells and symbiotic bacteria lead to dysfunctional humoral and cellular immune reactions in *Galleria mellonella* larvae (124). These findings suggest that *B. velezensis* infection interferes with the host’s gut microbiota, leading to dysbiosis, which, in turn, affects the host’s humoral immune response.

### 4.5 Conclusion

An increasing body of evidence suggests that specific bacteria can alter the composition of intestinal microbiota and play a key role in regulating the physiological state of hosts (125–127). In line with this, our findings indicate that the reconstruction of intestinal microbiota by Bv is the primary drive of growth and development inhibition in housefly larvae. Additionally, we found that Bv can affect insect humoral immunity by reducing phenoloxidase levels in the hemolymph. Furthermore, genome-wide analysis revealed that virulence factors carried by Bv could be another key factor contributing to housefly mortality. In summary, our research highlights that Bv profoundly influences insect growth and development by secreting certain virulence factors, disrupting gut microbiota and reducing immunity.

Currently, Bt is the most prevalently utilized insecticide in pest management. The Bt toxins have gained widespread acclaim due to their exceptional attributes, including high safety for humans and livestock, rapid biodegradability without persistent residues, and minimal environmental impact (128, 129). These advantages have led to the extensive adoption of Bt-based biological insecticides and Bt transgenic crops in the control of agricultural pests. Nevertheless, the emergence and escalation of pest resistance to Bt toxins pose significant challenges. This phenomenon severely restricts the large - scale cultivation of Bt transgenic crops and the sustainable application of Bt insecticides (130, 131). As a result, the development of novel, highly efficient alternative biological control agents has become an urgent and critical research priority in the field of pest management. Our bioassay results demonstrated that the *B. velezensis* (Bv) isolate exhibits potent larvicidal activity against housefly larvae. Notably, Bv possesses distinctive traits, including highly resilient structural features, a rapid growth phenotype, and high - yield characteristics, which render it eminently suitable for large - scale commercial production. Collectively, these characteristics highlight the promising potential of Bv as an alternative biocontrol agent for managing *Musca domestica* populations, as well as other insect pests. However, a few important aspects are still unanswered and need to be explored, i.e., (i) The safety of Bacillus velezensis to humans and non-target organisms, and its compliance with the requirements of green agriculture and sustainable development? (ii) How do specific virulence genes (e.g., ces, hlyIII) directly contribute to larval mortality? Functional validation (e.g., gene knockout) would strengthen claims. (iii) The tripartite interaction of host metabolism–*B.velezensis* infection–gut microbiota?

With advances in genomics, proteomics, and metabolomics, we will focus on developing and adopting novel integrated pest control technologies. These technologies along with other environment friendly pest control methods, will provide safer and more effective pest management. Meanwhile, studying bacteria-insect interactions could offer valuable new insights into the factors and mechanisms involved in human pathogenesis.

## Data Availability

The datasets presented in this study can be found in online repositories. The names of the repository/repositories and accession number(s) can be found in the article/**Supplementary Material**.
