# Supplementary material for: A taste of one’s own medicine: Bacillus velezensis isolated from adult housefly intestines demonstrates effective fly control
Source: Front Immunol. 2025 Sep 16;16:1575292. doi: 10.3389/fimmu.2025.1575292 (PMC12479530; doi:10.3389/fimmu.2025.1575292)
Supplement: Supplementary file 1 [file DataSheet1.pdf]

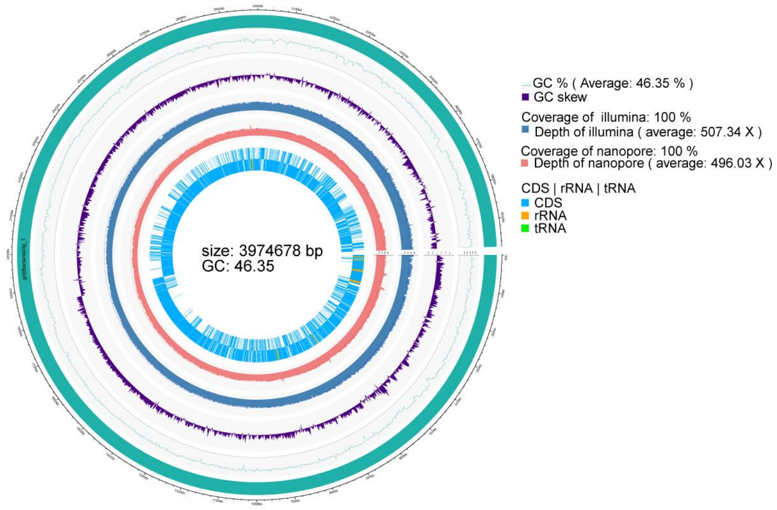

FIGURE S1 | Circular graphical representation of the genome and associated genes, including CDS on the forward strand, CDS on the reverse strand, RNA genes, GC content, GC skew, and sequencing depth.

Table S1 Parameter estimates by SPSS

Parameter	Estimate	Std. Eroor	*Z* test	Sig.	95% Confidence interval	
					Lower bound	Upper bound
Concentration	0.915	0.198	4.618	0.000	0.527	1.303
Intercept	-5.881	1.289	-4.564	0.000	-7.17	-4.593

Table S2 Chi-Square tests

	Chi-Square	df[a]	Sig.
Pearson goodness-of-fit test	2.144	5	0.829[a]

Table S3 95% Confidence limits (CL) for effective dose

Prob	Concentration	95% CL lower	95% CL upper
0.010	3.885	1.923	4.742
0.020	4.183	2.427	4.965
0.030	4.372	2.746	5.107
0.040	4.515	2.984	5.216
0.050	4.63	3.177	5.305
0.060	4.729	3.341	5.381
0.070	4.815	3.484	5.449
0.080	4.892	3.611	5.51
0.090	4.963	3.727	5.566
0.100	5.027	3.833	5.618
0.150	5.295	4.266	5.839
0.200	5.508	4.602	6.022
0.250	5.691	4.882	6.187
0.300	5.855	5.126	6.344
0.350	6.007	5.344	6.497
0.400	6.151	5.542	6.651
0.450	6.291	5.724	6.808
0.500	6.428	5.894	6.973
0.550	6.565	6.056	7.147
0.600	6.705	6.21	7.333
0.650	6.849	6.361	7.534
0.700	7.001	6.511	7.755
0.750	7.165	6.664	8.001
0.800	7.348	6.827	8.284
0.850	7.561	7.009	8.622
0.900	7.829	7.228	9.057
0.910	7.893	7.279	9.163
0.920	7.964	7.335	9.279
0.930	8.041	7.396	9.406
0.940	8.127	7.463	9.55
0.950	8.226	7.539	9.714
0.960	8.341	7.628	9.907
0.970	8.484	7.736	10.146
0.980	8.673	7.878	10.465
0.990	8.971	8.1	10.97

Table S4 Functions of the protein-coding genes of *B. velezensis* BV

Annotation database	No. of genes	Percentage of total (%)
KEGG	1228	32.47
Nr	3780	99.95
Uniprot	3773	99.76
GO	1081	28.58
COG	3072	81.23
Pfam	3329	88.02
Refseq	3760	99.42
Tigerfam	2180	57.64
